# Recovery from a Large Dose of Aconitina

**Published:** 1848-06

**Authors:** 


*Recovery from a large Dose of Aconitina.*—Dr. Golding Bird in relating to the Medical Society of London (Dec. 13th, 1847) some of the most remarkable facts connected with a case of poisoning by aconitina, stated that he was, from peculiar circumstances, not at present at liberty to allude to more than the actual symptoms presented whilst the patient was under his notice. He was anxious, however, to relate these, because they were not only very remarkable, but had not at present been recorded as the results of poisoning by the alkaloid in question; indeed, no case of this kind has been published, although several exist in which the roots and leaves of the aconite have accidentally produced death. There are few agents more likely to be employed by the secret poisoner, with greater certainty of escaping detection, than aconitina, the terrific power of which in destroying life, even in minute doses, is well known. A few days ago, Dr. Golding Bird was requested hastily to join a consultation on the case of a person holding an important position in society, and of very high intellectual attainments, who, under very peculiar circumstances, had swallowed some aconitina, which had been procured from one of the most respectable druggists in London, from a prescription of the patient's own writing, he having devoted some part of his time to the study of physic and chemistry, as a part of his very extended education. From collateral evidence, it appeared probable that almost immediately after taking the poison, the person must have fallen, and struck his head a severe blow against some furniture in the room. The poison, or the blow, or both, must have produced immediate violent vomiting, as the room was flooded with vomited matter. How soon he was discovered after this event was not clear, from the peculiar circumstances in which he was placed. An excellent and most intelligent surgeon, and shortly after a physician, were called to him; and about two o'clock in the afternoon, which must at least have been eight hours after taking the poison, Dr. G. Bird met these gentlemen. The patient was then fearfully collapsed; the surface cold and sweating; quite pale; the heart's action scarcely perceptible; pupils acting to light; no paralysis whatever, either of sensation or motion. Notwithstanding the intense exhaustion, the intellect was unimpaired. The most prominent symptom was the repeated and terrific vomiting of a brownish fluid. This vomiting was, however, peculiar, and perhaps hardly deserved that title, the patient really being seized with a kind of spasm, during which he convulsively turned upon his abdomen, and with an intense contraction of the abdominal muscles, he jerked out, as it were, the contents of his stomach, with a loud shout, depending, apparently, on the sudden contraction of the diaphragm,. These exhausting and distressing symptoms had occurred every minute or two. On attempting to make him swallow any fluid, a fearful spasm of the throat took place, producing the distressing effects so well known in the hydrophobia from the bites of rabid animals: this was not produced by the sight of water, but the convulsive movements of the body, and emptying of the stomach, were excited by abruptly touching him. He was placed in a hot bath, aftewards removed to bed, covered with blankets, a large mustard poultice applied to the scrobiculus cordis, and an enema of turpentine administered. He remained in much the same state, the sedative effects of the poison on the heart gradually lessening, so that at nine o'clock the pulse was easily perceptible, although weak; the hydrophobic spasms were, however, then produced by every attempt to swallow, so that none of the medicines suggested for him could be made use of. It was therefore determined merely to administer enemata of beef-tea, with the yolk of an egg, and ten grains of laudanum, chiefly with the intention of procuring rest and giving support. He had a fearful night of exhaustion and spasm; intellect perfect, and even vivid, so as to enable him, whenever he recovered a little from his exhaustion, to carry on a conversation in the French language with an excellent and distinguished clergyman, who shared the task of soothing and watching him, with his zealous surgeon. After this hard struggle with death, this very remarkable man emerged from the effects of the poison: and at two o'clock on the following day, when another consultation was held, he was regarded as convalescent. Dr. Golding Bird drew attention to the fact, that although at least *two grains and a half* of aconitina had been swallowed, yet that the majority of the poison must have been got rid of during the vomiting which followed the injury to the head, resulting from the fall. He also pointed out the remarkable train of symptoms developed, especially the convulsive vomiting and imperfect hydrophobia, as possibly being characteristic of the effects of the poison. For although they differed importantly from the effects of aconite root, with the exception of the sedative influence on the heart, still such a difference becomes intelligible when it is recollected that a pure alkaloid often differs materially in its physiological action from that of the plant from which it is an extract, as shown remarkably in the case of the alkaloid conia.—*Lond. Med. Gaz.*

